# Dynamics of Tree Species Diversity in Unlogged and Selectively Logged Malaysian Forests

**DOI:** 10.1038/s41598-018-19250-z

**Published:** 2018-01-18

**Authors:** Ken Shima, Toshihiro Yamada, Toshinori Okuda, Christine Fletcher, Abdul Rahman Kassim

**Affiliations:** 1 Graduate School of Integrated Arts and Sciences, Hiroshima University, 1–7–1 Kagamiyama, Higashi–Hiroshima, 739–8521 Japan; 2 Forest Research Institute of Malaysia, 52109 Kepong, Selangor, Malaysia

## Abstract

Selective logging that is commonly conducted in tropical forests may change tree species diversity. In rarely disturbed tropical forests, locally rare species exhibit higher survival rates. If this non–random process occurs in a logged forest, the forest will rapidly recover its tree species diversity. Here we determined whether a forest in the Pasoh Forest Reserve, Malaysia, which was selectively logged 40 years ago, recovered its original species diversity (species richness and composition). To explore this, we compared the dynamics of secies diversity between unlogged forest plot (18.6 ha) and logged forest plot (5.4 ha). We found that 40 years are not sufficient to recover species diversity after logging. Unlike unlogged forests, tree deaths and recruitments did not contribute to increased diversity in the selectively logged forests. Our results predict that selectively logged forests require a longer time at least than our observing period (40 years) to regain their diversity.

## Introduction

Selective logging is used commonly for commercial logging in Southeast Asian tropical forests, and it causes forest degradation, such as decreased carbon stocks, lower canopy complexity^1,2^, and reduced faunal species diversity^3^. Selective logging may change the original species composition of trees via the removal of large trees and the subsequent recruitment of early successional tree species. However, the impacts of selective logging on tree biodiversity are still controversial. Putz *et al*.^4^ showed that trees species richness remain unchanged just after selective logging, while Gibson *et al*.^5^ showed that selective logging decreased plant biodiversity. Furthermore, little is known about the long–term impact of selective logging on tree biodiversity, because most studies have examined forests only shortly (within 12 years) after logging^5^. Here we demonstrate a long–term impact of selective logging on tree species biodiversity by comparing two tropical forest plots in Malaysia, one of which was set in an unlogged forest, while the other was set in a selectively logged forest.

Tree species diversity (species richness, composition and evenness) should change with time. Provided that there are no interspecific differences in reproductive and survival rates, random tree deaths and the recruitment of random species will lead to stochastic fluctuations in the populations of all tree species, some of which will go extinct^6^. Therefore, tree species diversity in a forest will decrease with time. However, given that death and recruitment will not occur randomly, and if the recruitment of locally rare species and/or the survival of locally rare species increases, the erosion of species diversity by stochastic processes will be prevented. Wills *et al*.^7^ discovered that locally rare species have higher survival rates than abundant species in many tropical forests that have been subjected to few human disturbances.

Three ecological models have been proposed that explain why a species increases its survival and/or recruitment rates when it becomes locally rare. The first model is the Janzen–Connell hypothesis^8,9^. This model states that species diversity is maintained by density–dependent interactions between hosts and specialized pathogens, herbivores, or predators. The Janzen–Connell model predicts that species diversity should increase with time because of the selective removal of abundant species by pathogens and predators. The second model is the niche complementarity hypothesis^10–12^. This model predicts that individuals compete more intensively with conspecific individuals than with individuals of other species. Thus, locally abundant species are at a relative disadvantage because they are subjected to more conspecific competition. The final model is the facilitation hypothesis^13–15^. This model is similar to the niche complementarity hypothesis. Facilitation means that locally rare species are advantaged because an individual benefits a neighboring individual of another species. If any of these three models is correct, a forest ecosystem will maintain or even increase its local species diversity with time, as has been observed in many tropical forests^7^.

If the density–dependent mechanism is correct, species diversity, after a temporary decrease in response to damage, will recover quickly to its prior level^7^. However, this idea has never been tested in any tropical rainforest. Here we addressed the following three questions using a dataset derived from long–term observations of forests: (1) Does a forest that was logged selectively 40 years ago recover its original tree species composition? (2) How does tree biodiversity change with time in old, selectively logged and unlogged forests? (3) How do tree death and recruitment contribute to tree species richness in selectively logged and unlogged forests? These questions were linked with the aforementioned prediction^7^ based on long–term forest dynamics data obtained from unlogged tropical forests.

## Methods

### Study sites and field data

In this study, we used tree census data that were derived from plots established in the Pasoh Forest Reserve (latitude 2°59′N, longitude 102°18′E), Negri Sembilan, Peninsular Malaysia^16^. The average annual rainfall at Pasoh–Dua (latitude 2°56′N, longitude 102°18′E), approximately 6 km south of the reserve, from 1974 to 2009 was 1,842 mm. In the study area, the soil type develops mainly from shale, granite, and fluviatile granite alluvium parent materials^17^. The topography consists mainly of flat alluvial areas, with smaller expanses of swales, riverine areas, and gently rolling hills^18,19^. The vegetation type in the reserve is a lowland dipterocarp forest that is dominated by Dipterocarpaceae species^20–22^.

The unlogged forest has never been disturbed anthropogenically, and it is a representative example of the lowland forests of south–central Peninsular Malaysia^23,24^. However, in the Pasoh Forest Reserve, a certain portion of the forest was logged selectively under the Malayan Uniform System (MUS) in the 1950s, 1960s, and 1970s. The MUS cut all trees with a DBH ≥45 cm (all species). The detail information on the MUS was found in Wyatt-Smith^25^.

A 50–ha (north–south 500 m × east–west 1,000 m) plot in the center of an unlogged forest was established (hereafter referred to as the “unlogged forest plot”)^26^. Additionally, a 6–ha (north–south 200 m × east–west 300 m) plot was set in a forest that was logged selectively in 1958 (hereafter referred to as the “logged forest plot”)^1,27^. A tree census of the unlogged forest plot was conducted at 5–year intervals beginning in 1985, while a tree census of the logged forest was conducted at 2–year intervals from 1998 to 2012. In both plots, all tree plants with a DBH ≥ 1 cm were identified, measured, and tagged, and their positions were mapped. We measured local species diversity on a subplot basis.

To minimize the impacts of pre–logging differences on the differences in tree species composition between the two plots, both plots were set as close as possible (the distance between the plots is 3 km) to each other. In the reserve, topographic and edaphic conditions affect the species composition^19,28,29^. To remove the edaphic–effect–induced difference in species composition between the plots as much as possible, we used and analyzed data derived from parts of the plots where the soil type was identical. We subdivided the plots into subplots (10 × 10 m). Almost all the logged plot (90%; 5.4 ha) were covered by the hilly area. However, the unlogged plot accounted only for 37.3% (18.6 ha) of the hilly area. Then, we used only the hilly parts of both plots to minimize the topographic effects on species composition. Yet, some differences between the plots remained. For example, the logged plot was located close (0.5 km) to an oil palm plantation, while the unlogged plot was farther (1.5 km) from the oil palm plantation.

In the unlogged forest plot, all census data were used because all the trees in the plot were identified at the species level. However, in the logged plot, we could not use the tree census data, because we could not identify tree species because of leaf fall (253 trees) or we could identify them only to the genus revel (104 trees). Therefore, we used only 34,162 (98.7%) of the trees in the analysis.

### Data analysis

#### Tree species diversity

We quantified local species diversity based on the 10 m × 10 m subplots. To quantify species diversity, we used a rarefaction index^30^. The rarefaction index is a measure of the expected number of species in a 10 m × 10 m subplot of an arbitrary number of individuals (10 trees in this study). Most other commonly used species diversity indices, such as the Shannon–Weaver species diversity index, are influenced by sample size^7,31,32^. However, the rarefaction index exhibits little correlation with sample size^7^. Species diversity is influenced by both the species number and species evenness of a community^33^. To reveal how species number and evenness contribute to species diversity, we obtained the species number (richness) and evenness separately as follows. We calculated Pielou’s evenness index, *J′*^33^, to evaluate species evenness using the following equation:

1 $$J^{\prime} =H^{\prime} /H{^{\prime} }_{max}$$

where *H′* is the Shannon–Weaver species diversity index and *H′*_*max*_ is the maximum level of species diversity possible within a forest community. Note that unlike the rarefaction index, both species number and *J′* are influenced by sample size.

To reveal the temporal transition of species diversity, we calculated the species number and evenness, as well as the rarefaction index, in all census years. We determined the correlation between species diversity and time using Pearson’s product–moment correlation coefficient. The change in species diversity between two consecutive censuses was examined as follows. The rarefaction index within a subplot in each census year was subtracted by the rarefaction index of the subplot in the previous census year. If this value is positive, species diversity increased during this time, while a negative value indicates that species diversity decreased. We compered the rarefaction index every 5 years for the unlogged plot and for 1998–2002 (4 years), 2002–2006 (4 years), and 2006–2012 (6 years) in the logged plot because we could not detect a small change in the rarefaction index at shorter (2–year) periods. These time intervals are long enough to see tree species richness dynamics for tree over 1 cm DBH. To examine the difference in the rarefaction index between the plots, we compared the mean rarefaction index in 2000, the only year in which the census was conducted on both plots, using a *t*–test.

#### Effect of mortality and recruitment on species diversity

Changes in species diversity caused by mortality and recruitment may have occurred between the censuses. Therefore, we quantified the effects of mortality and recruitment on species diversity separately as follows. We denoted the tree community vector (number of trees per species) at the *i*th census by *T*_*i*_ and those of recruited and dead trees between the *i*th and (*i*−1) censuses by *R*_*i*_ and *D*_*i*_, respectively. T_*i*_ is related to *R*_*i*_, *D*_*i*_, and *T*_*i*−1_ (the tree community vector at the previous census) as follows^34^:

2 $${T}_{i}={T}_{i-1}+{R}_{i}-{D}_{i}$$

To examine the effect of mortality and recruitment on species diversity between the *i*th and (*i*−1)th censuses, we used two indices from Yamada *et al*.^34^:

3 $${{\rm{ED}}}_{i}={\rm{rarefaction}}\,({T}_{i-1}-{D}_{i})-{\rm{rarefaction}}\,({T}_{i-1})$$

4 $${{\rm{ER}}}_{i}={\rm{rarefaction}}\,({T}_{i-1}+{R}_{i})-{\rm{rarefaction}}\,({T}_{i-1})$$

For both indices, the values of ED_*i*_ and ER_*i*_ represent the effects of mortality and recruitment on species diversity. We calculated ED_*i*_ and ER_*i*_ for each subplot every 5 years for the unlogged plot and for 1998–2002, 2002–2006, and 2006–2012 for the logged plot. When ED_*i*_ and ER_*i*_ are positive, species diversity was increased by mortality and recruitment, respectively, during the period. When ED_*i*_ and ER_*i*_ are negative, species diversity was decreased by mortality and recruitment, respectively. We calculated the mean ED_*i*_ and ER_*i*_ per subplot, and the 95% confidence interval. If the 95% confidence interval did not include 0.0, the effect was considered to be statistically significant. All statistical analyses were performed using R 3.3.1^35^.

#### The expected number of species in both plots

**Figure 1:**
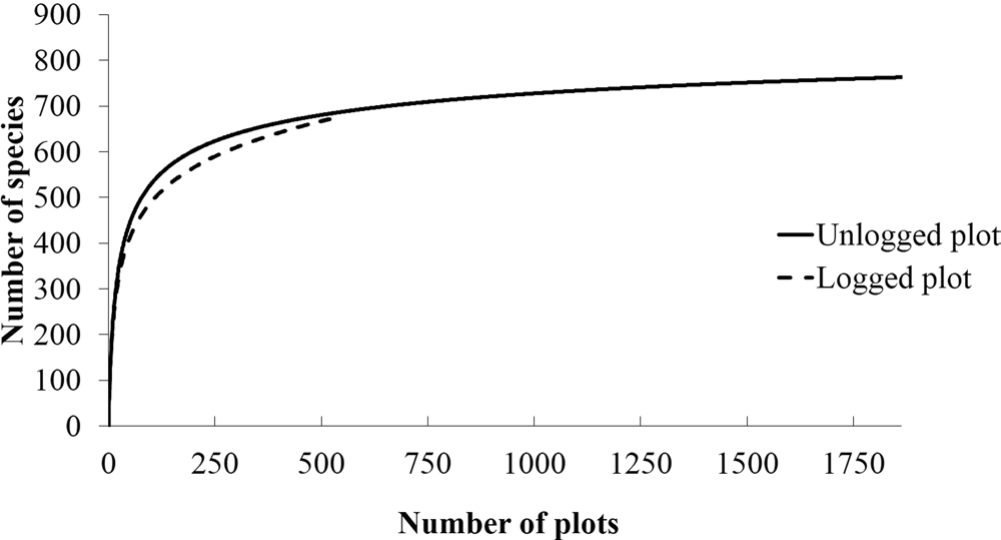
Species–area curves in the unlogged and logged plots in the Pasoh Forest Reserve. Species–area curves based on 10 × 10 m subplots were produced by a Jackknife1 estimation^36–38^ using the data from the 2000 census. The solid and dotted lines represent the unlogged (18.6 ha) and logged (5.4 ha) plots, respectively. To avoid a sampling effect induced by the order of the subplots on the species–area curve, we ran 100 trials to construct the species–area curve by randomly changing the order of the subplots, and we determined the average species–area curve over the 100 trials and the 95% confidence limits for the average species–area curve.

We calculated the proportion of the number of observed common species that were present in both plots relative to all species using tree census data from 2000, which is the only year when the tree census was conducted on both plots. We also calculated the expected number of common species that should be present if both plots were set in the unlogged forest as follows. First, we drew species–area curves based on the unlogged plot data using the Jackknife1^36–38^ method using the following equation:

5 $${S}_{jack1}={S}_{obs}+Q\{(m-1)/m\}$$

where *S*_*obs*_ is the number of observed species; *Q* is the number of species in a subplot (10 × 10 m); and *m* is the number of observed trees. The species–area curves resulted in an increasing number of species in each subplot. To avoid a sampling effect induced by the order of the subplots on the species–area curve, we ran 100 trials to generate the species–area curves by randomly changing the order of the subplots, and we determined the average species–area curve and the 95% confidence limits for the average species–area curve over the 100 trials. Next, we calculated the expected number of species that should be present in the unlogged plot (18.6 ha) and the logged plot (5.4 ha) based on the average species–area curve, which resulted in 763 species per 18.6 ha and 686 species per 5.4 ha in the respective plots (the solid line on Fig. 1). Then, we calculated the proportion of these species numbers to the number of species in the stationary phase (asymptotic period) in the unlogged forest that was expected from the species–area curve (815 species). Therefore, we predicted that 93.6% (763 species/815 species) and 84.2% (686 species/815 species) of the species should be present in the 18.6 ha and 5.4 ha plots, respectively. Finally, we calculated the expected number of overlapping species between the two plots as the product of these proportions (78.8%), which resulted in 642 (815 species × 0.788) expected common species. The difference in the species composition between the unlogged plot and the logged plot was tested by comparing the proportion of observed common species and that of expected common species (78.8%) using Fisher’s exact test for count data. These analyses were performed by EstimateS^39^.

### The proportion of early successional species

We compared the proportion of trees comprising early successional species to all trees between the plots. If the proportion of early successional species is higher in the logged plot than the unlogged plot, it is highly likely that the impacts of the logging will be retained^40^. The identification of early successional species was determined as described by Davies *et al*.^41^; they listed typical early successional species of the top 30 abundance in the unlogged plot. Briefly, the list has three *Macaranga* species, which is the most typical early successional species. The listed species differs ecological traits each other. For example, the list contains long-lived species like *Alstonia angustiloba* Miq. and *Dyera costulata* (Miq) Hk. *f*. The proportion of early successional trees to all trees was calculated. We tested the difference in the proportion of early successional species between the plots using Fisher’s exact test for count data.

## Results

### Difference in the species composition between the plots

In the tree census year of 2000, 763 species were present in the 18.6–ha hilly area of the unlogged plot, and 676 species were present in the 5.4–ha hilly area of the logged plot. Among the present species, 178 (20.7%) were present exclusively in the unlogged plot, and 89 (10.4%) were present exclusively in the logged plot. Therefore, the remaining 587 species (68.7%) were present in both plots. Additionally, the proportion of the expected number of common species between the plots was 78.8% based on the species–area curve, and this proportion was higher than the observed proportion of common species (68.7%) (Fisher’s exact test for count data, *p* < 0.01).

The proportion of early successional species in the unlogged forest in 2000 was 3.29% (early successional species comprised 3,458 trees, and the total species comprised 105,193 trees) in the unlogged forest, while this proportion in the logged forest was 4.29% (early successional species comprised 1,328 trees, and the total species comprised 30,940 trees). The proportion of early successional species in the logged forest was higher than that in the unlogged forest (Fisher’s exact test for count data, *p* < 0.01).

We compared the species diversity between the plots using the tree census data of 2000. The average numbers of species per subplot were 43.0 and 42.5 in the unlogged and logged plots, respectively, which did not differ (Welch’s *t*–test, *t* = 1.00, *p* = 0.32). The rarefaction indices per subplot were 9.43 and 9.28 in the unlogged and logged plots, respectively, and they differed (Welch’s *t*–test, *t* = 7.89, *p* < 0.01).

According to the comparison between the plot–specific species–area curves, it is unlikely that there is a major difference between the unlogged and logged forests at larger scales (over 5 ha) (Fig. 1). The number of species was higher in the unlogged forest than the logged forest at medium scales (1–4 ha); however, the 95% confidence intervals overlapped, indicating that there was no difference in the number of species.

### Dynamics of species diversity

**Figure 2:**
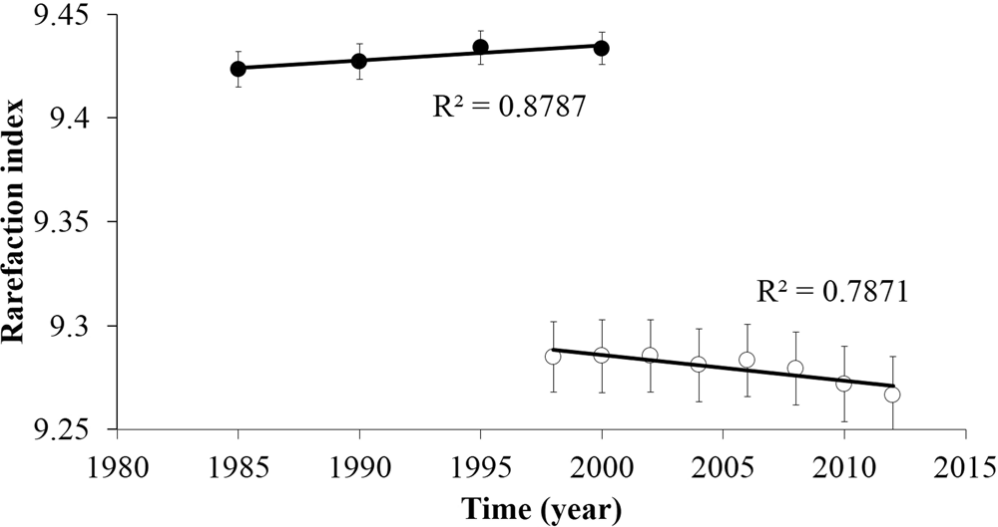
Time–trend change in the tree species diversity in the unlogged and logged plots in the Pasoh Forest Reserve. Time–trend change in the rarefaction index per subplot (10 m × 10 m) from 1985–2000 in the unlogged plot (filled symbols) and from 1998–2012 in the logged plot (open symbols). Circles represent the average value per subplot. Bars represent standard errors.

**Figure 3:**
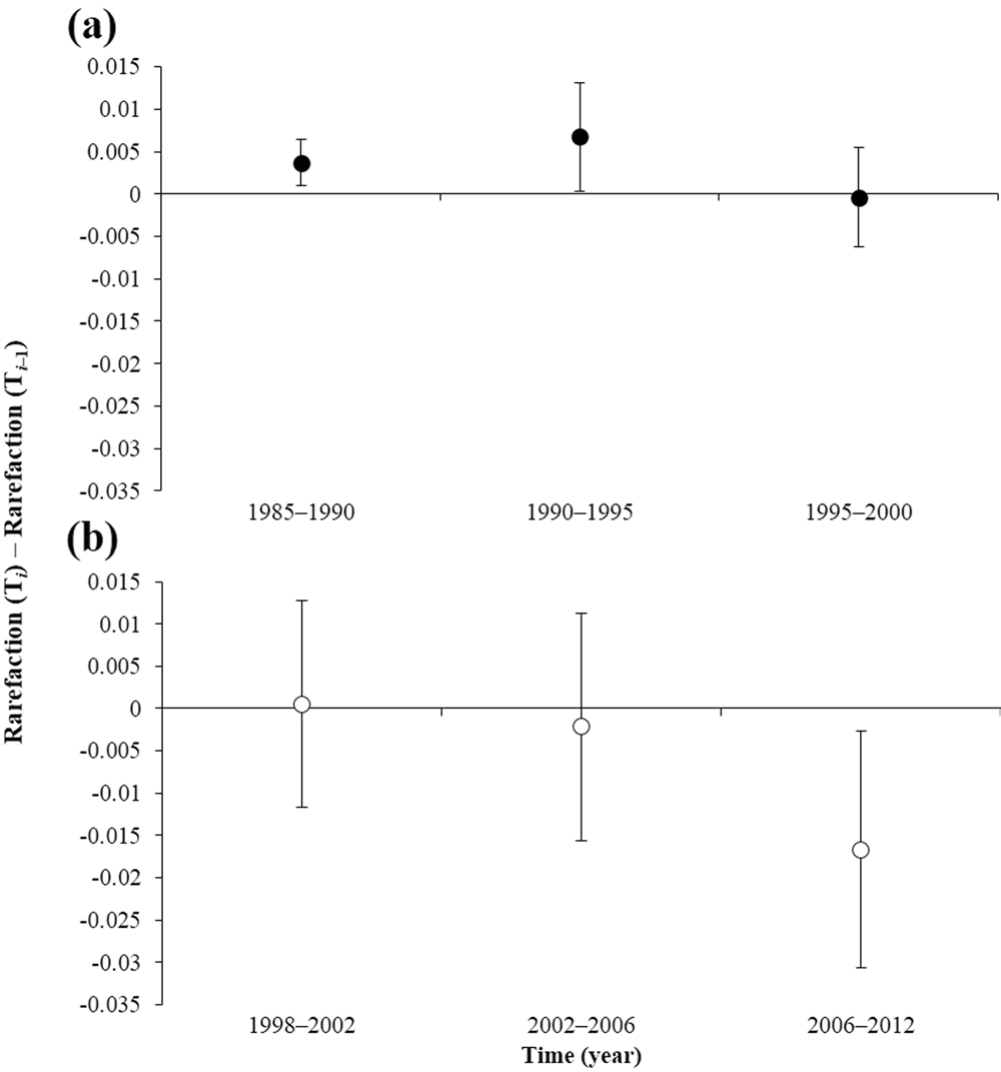
Differences in the rarefaction index in each census year from that of the previous census year in the unlogged and logged plots in the Pasoh Forest Reserve. Differences in the rarefaction index in each census year from that of the previous census year in the unlogged plot (**a**) and the logged plot (**b**). Positive values show that the species diversity increased, and negative values show that the species diversity decreased. Circles represent mean values. Bars represent 95% confidence intervals for the difference in the rarefaction index per subplot.

The rarefaction index in the unlogged plot for 1985–2000 increased over time, but it was not correlated with time, possibly because of the small sample size (*n* = 4) (Pearson’s product–moment correlation coefficient, *R*^2^ = 0.879, *p* = 0.06) (Fig. 2). In contrast, the rarefaction index in the logged plot for 1998–2012 showed a negative correlation with time (Pearson’s product–moment correlation coefficient, *R*^2^ = 0.787, *p* < 0.01). Increased and decreased species diversity over time could be observed by examining the difference in the rarefaction index between two consecutive census periods. The species diversity in the unlogged plot for 1985–1990 and 1990–1995 increased significantly, while a non–significant increase was observed for 1995–2000 (Fig. 3a). In contrast, the change in species diversity in the logged forest during 1998–2002 and 2002–2006 was not significant, while that for 2006–2012 showed a statistically significant decrease (Fig. 3b). The species evenness, *J*′, in both plots tended to increase with time (Pearson’s product–moment correlation coefficient, *R*^2^ = 1.00, *p* < 0.01 for the unlogged plot, and *R*^2^ = 0.999, *p* < 0.01 for the logged plot). The number of species in both the unlogged and logged plots showed decreases (Pearson’s product–moment correlation coefficient, *p* < 0.01).

### The effect of mortality and recruitment on tree species diversity

**Figure 4:**
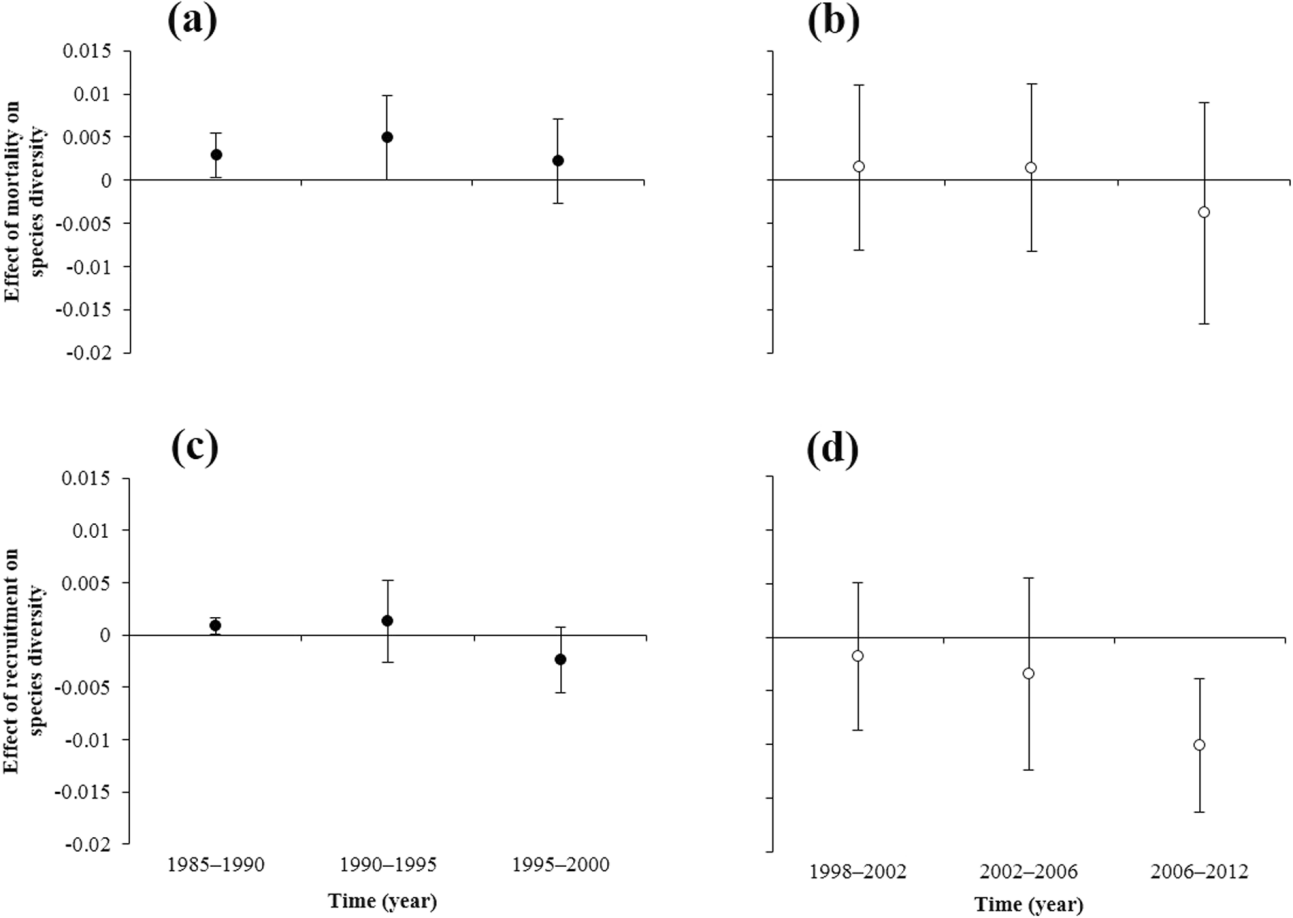
The effect of mortality (ED) and recruitment (ER) on species diversity in the unlogged and logged plots in the Pasoh Forest Reserve. The effect of mortality on species diversity (ED) in the unlogged plot (**a**) and the logged plot (**b**). The effect of recruitment on species diversity (ER) in the unlogged plot (**c**) and the logged plot (**d**). ED_*i*_ is rarefaction (*T*_*i*−1_ − *D*_*i*_) minus rarefaction (*T*_*i*−1_). ER_*i*_ is rarefaction (*T*_*i*−1_ + *R*_*i*_) minus rarefaction (*T*_*i*−1_). Circles represent the mean ED and ER values per subplot. Bars represent 95% confidence intervals.

The average values of ED in the unlogged plot were positive for all periods, and the values were higher than 0.0, except for the 1995–2000 period (Fig. 4a). In the unlogged forest, mortality had a positive effect on tree species diversity as well. However, the 95% confidence interval of the average ED value in the logged plot included 0.0 for all the census periods (Fig. 4b), showing that mortality in the logged forest did not have a statistically significant effect on tree species diversity. In short, mortality increased species diversity in the unlogged forest, but not in the logged forest.

The average ER values in the unlogged plot for 1985–1990 were positive, and the 95% confidence interval did not include 0.0 (Fig. 4c), while those for 1990–1995 and 1995–2000 included 0.0. Thus, in the unlogged forest, recruitment had a positive to neutral effect on species diversity. In contrast, when examining the logged plot, the average ER values were negative for all the census periods, and the 95% confidence interval for 2006–2012 excluded 0.0. Thus, in the logged forest, recruitment had a negative to neutral effect on species diversity (Fig. 4d).

When the species diversity increased in the unlogged forest during 1985–1990 and 1990–1995 (Fig. 3a), the effect of mortality on tree species diversity during these periods was also positive. In 1995–2000, when the species diversity was unchanged, mortality has a neutral effect on tree species diversity. In addition, during 1985–1990, recruitment had a positive effect on tree species diversity. These combinations of the effects of mortality and recruitment on species diversity could explain the dynamics of species diversity in the unlogged forest plot.

In the logged forest plot, species diversity did not change for 1998–2002 and 2002–2006 (Fig. 3b), and both mortality and recruitment had neutral effects on species diversity for these periods. The species diversity for 2006–2012 decreased (Fig. 3b). During the same period, mortality had a neutral effect on species diversity, while recruitment had a negative effect.

## Discussion

Our results showed that the species composition differed between the unlogged and logged forests long (40 years) after logging operations ceased. Early successional species were supposed to increase just after logging because logging opens forest canopies and to decrease over time. We expected that the change in species composition in the logged forest would be similar to that in the unlogged forest over time^42^. However, our results showed that the proportion of early successional trees to all trees in the logged forest did not reach the level of that in the unlogged forest, suggesting that 40–50 years is not sufficient for a forest to recover its original species composition after logging. This differs from observations in a mixed dipterocarp rainforest site in Kalimantan, Indonesia, which suggested that the seeding of early successional species was almost eliminated 12 years after a logging operation^43^. One possible explanation for the difference between these results may be the time lag for the elimination of early successional species. Perhaps the seedling recruitment of early successional species starts to become limited shortly after logging, but the elimination (death) of trees of long-lived early successional species takes longer. In a Japanese natural forest, the elimination of early successional species took more than 50 years after their recuitment^44^. But we also have to pay attention to idea that the species composition difference might have been produced by other factors than logging since we could not eliminate pre-logging difference compeletely as we described in “Methods”.

The unlogged and logged forests showed opposite patterns of dynamics of tree species diversity. The species diversity in the unlogged forest increased with time, while that in the logged forest decreased with time. The change in the species diversity with time could be caused by a change in species composition through death and recruitment. Our further examinations showed that the death and recruitment of trees contributed differently to the change in species diversity between the forests. Tropical forests commonly exhibit a pattern in which a higher conspecific local density increases the local mortality of conspecific individuals^7^, thereby apparently increasing local species diversity by increasing species evenness. We also showed that deaths of trees increased the species diversity in the unlogged forest. There are three important models that seek to explain density–dependent tree deaths: the Janzen–Connell hypothesis^8,9^, the niche complementarity hypothesis^10–12^, and the facilitation hypothesis^13–15^. Although our data strongly support the existence of density–dependent mortality and that it increased local species diversity in the unlogged forest, we still do not know which model is most appropriate for the Pasoh Forest Reserve.

We looked closely at the effect of mortality on species diversity. Wills *et al*.^7^ found a negative, density–dependent mortality in tropical forests, including the Pasoh Forest Reserve. In this process, species diversity increases with time in this tropical forest.

In contrast, mortality had a neutral effect in the logged forest. Wills *et al*.^7^ predicted that ecosystems that have lost their species diversity via temporary damage may recover their former species diversity rapidly via negative–density–dependent mortality. However, this prediction does not explain our results, because the effect of mortality in the logged forest did not conform to the predictions of Wills *et al*.^7^ Negative–density–dependent mortality might be diminished by logging operations, although the underlying mechanisms are unknown.

Next, we examined the effect of recruitment on species diversity. Wills *et al*.^7^ showed that the higher the conspecific local density, the higher the local recruitment of conspecific individuals. Because this mechanism increases the dominance of a species, it must decrease local species diversity. Our results in the logged forest agrees with the prediction by Wills *et al*.^7^. However, in the unlogged forest, recruitment had positive to neutral effects on species diversity, which contradicts the findings of Wills *et al*.^7^. The mechanism by which recruitment increased the species diversity in the unlogged forest is still unknown; thus, future studies are needed to examine this issue.

We assumed that the difference in the species dynamics between the unlogged and logged forest was brought by logging but there might be a factor other than logging that accounts for. In this study, when we established the unlogged and logged plots, we paid great attention to ensure that the plots were similar in terms of their topography and soil. However, we cannot exclude completely the possibility that the pre–logging status differed between the plots. Wild pigs (*Sus scrofa*) are known to damage trees^45,46^. The impact of pigs on trees was severe in the Pasoh Forest Reserve; according to Ickes and Thomas^47^, in the unlogged forest, which seems to have lower wild–pig activity than the logged forest, wild pigs caused 29% of the total tree mortality in the 1–2–cm DBH size class. The wild pigs cut trees in this size class to make their nests. In the Pasoh Forest Reserve, wild pig activities were higher in the logged plot, which was located closer to an oil palm plantation than the unlogged plot, because pigs are known to feed on oil palm seeds^47,48^. Thus, we expect that the impacts of wilds pigs on trees were also higher in the logged plot. They cut and kill trees to make nests. While the tree preference of wild pigs is independent of the tree species density, wild pigs may search for preferred tree species to build their nests. Hence, the large disturbances by wild pigs in the logged forest might have obscured the density–dependent mortalities that were observed in the unlogged forest.

The abovementioned oil palm plantation induced difference in wild pig activity is a typical edge effect, which is the impacts on a forest ecosystem permeating from the forest edge. Edge effects are known to severely impact forest dynamics in many tropical forest^49,50^. For example, tree mortality rates were increased by the edge effects in an Amazon forest^49^. Typically, penetrated distance of edge effects is 1.0 km for invertebrates, vertebrates and trees in tropical forests^50^. Since the logged and unlogged plots were 0.5 km and 1.5 km away from the forest edge (oil palm plantations), respectively, the impacts of the edge effects might predominately work in the logged plot and might cause the difference in species diversity between them.

Species composition after logging is believed to recover to its prior pre–logging operation level^42^. Based on the findings from unlogged forests^7^, we could deduce that tropical forests that have lost species diversity by selective logging will recover species diversity rapidly. But our study did not meet this. We showed there were clear differences in species diversity and species composition between the unlogged and logged forests 40 years after logging. In addition, the species diversity in the logged forest decreased with time during our study period. These results suggest that it is not clear whether the species composition of the logged forest recovered to the level of that preceding the logging. At least, we can certainly say that 40–50 years are not sufficient for species diversity to recover after selective logging.

However as mentioned above, in our plot design, it is difficult to disengage whether the observed slow recovery rate of species diversity owes to either logging or to the edge effects associated with the proximity to oil palm plantations. Perhaps, the combination of logging and edge effects may result in the slow recovery rates. Thus, we have to acknowledge that it is still unclear whether our results are applicable to other logged tropical forests; therefore, we need to confirm the applicability of our results with the plot design that independently treats logging and edge effects.

A recent study revealed the importance of managing a logged forest for biodiversity^4^. While we agree that good forest management not only for the future timber production but also for the recovery of species diversity after logging is essential, the protection of unlogged forests is very important for the conservation of invaluable tropical biodiversity^5^.
